# Temporary Fecal Diversion in the Management of Colorectal and Perianal Crohn's Disease

**DOI:** 10.1155/2015/286315

**Published:** 2015-01-11

**Authors:** Rudolf Mennigen, Britta Heptner, Norbert Senninger, Emile Rijcken

**Affiliations:** Department of General and Visceral Surgery, University Hospital Muenster, Albert-Schweitzer-Campus 1, 48149 Muenster, Germany

## Abstract

*Aim*. To evaluate the results of temporary fecal diversion in colorectal and perianal Crohn's disease. *Method*. We retrospectively identified 29 consecutive patients (14 females, 15 males; median age: 30.0 years, range: 18–76) undergoing temporary fecal diversion for colorectal (*n* = 14), ileal (*n* = 4), and/or perianal Crohn's disease (*n* = 22). Follow-up was in median 33.0 (3–103) months. Response to fecal diversion, rate of stoma reversal, and relapse rate after stoma reversal were recorded. *Results*. The response to temporary fecal diversion was complete remission in 4/29 (13.8%), partial remission in 12/29 (41.4%), no change in 7/29 (24.1%), and progress in 6/29 (20.7%). Stoma reversal was performed in 19 out of 25 patients (76%) available for follow-up. Of these, the majority (15/19, 78.9%) needed further surgical therapies for a relapse of the same pathology previously leading to temporary fecal diversion, including colorectal resections (10/19, 52.6%) and creation of a definitive stoma (7/19, 36.8%). At the end of follow-up, only 4/25 patients (16%) had a stable course without the need for further definitive surgery. *Conclusion*. Temporary fecal diversion can induce remission in otherwise refractory colorectal or perianal Crohn's disease, but the chance of enduring remission after stoma reversal is low.

## 1. Introduction

Despite modern medical therapies, refractory colorectal or perianal Crohn's disease still mandates surgical interventions. For colorectal Crohn's disease, this usually means colectomy with ileorectal anastomosis or terminal ileostomy, or, in cases of severe rectal inflammation or complex perianal manifestations, proctocolectomy with terminal ileostomy. However, these operations have relevant and irreversible consequences that may not be acceptable especially for young patients.

The temporary fecal diversion by means of a loop ileostomy or colostomy is a much less invasive and mutilating option in these situations. Several authors have already reported astonishing rates of clinical remissions of colorectal Crohn's disease following stoma creation in the 60s and 70s [1–4]. In these early reports, the authors primarily employed stoma creation to control the inflammatory process and to ameliorate the general condition and the nutritional status of their patients before proceeding to definite surgical resections as mentioned above (like a “bridge to surgery”). In the 80s and 90s, most authors still did not recommend reversal of these stomas as they saw little chance of enduring remission once the intestinal continuity was restored [5, 6], while only few authors proposed reversal of these stomas in case of clinical remission [7].

Nowadays, the value of temporary fecal diversion is still under discussion, and only relatively few reports exist on outcomes of this concept reporting variable success rates [8–12].

The aim of this study was to answer the following questions.

1. Does temporary fecal diversion induce remission in otherwise refractory cases of colorectal and perianal Crohn's disease?
2. Are stomas really reversed as initially intended (indeed “temporary”)?
3. What is the clinical course after stoma reversal?
4. Does the strategy of temporary fecal diversion have a place in the management of colorectal and perianal Crohn's disease?

## 2. Patients and Methods

The setting is a retrospective single center study at our tertiary referral center (university hospital). The study was approved by the Ethics Committee of Muenster University, and informed consent was obtained for all interventions. We reviewed our prospectively maintained inflammatory bowel disease database to identify patients with colorectal or perianal Crohn's disease who received a fecal diversion as surgical therapy (without synchronous colorectal resections); fecal diversions had to be intended as “temporary” with the perspective of stoma reversal. Between 02/2003 and 12/2012, we identified 33 consecutive patients. Four patients were excluded as relevant clinical data and follow-up were missing, so 29 patients were included in the study.

Decisions to establish temporary fecal diversion (instead of definitive surgical resections) were made on an individual basis after discussion with the patients. All included patients were refractory to medical therapy. Frequent arguments for this procedure were young age of the patient, patient's refusal of definitive surgical resections, or the perspective to start anti-TNF-alpha therapy that was prevented by abscesses, perianal sepsis, or bad general condition. Creation of a loop ileostomy was the standard procedure for fecal diversion; however, in four patients, a loop colostomy was created. Stoma reversal was done by interrupted hand suture as standard procedure.

Demographic data, duration of disease, indication for stoma creation, clinical course after stoma creation including endoscopy, diagnostic imaging, and clinical parameters, stoma reversal, clinical course following stoma reversal, and medication (especially immunosuppressives and anti-TNF-alpha antibodies) were recorded. For patients who were not seen in our outpatient clinic on a regular basis, questionnaires were sent out to the responsible gastroenterologist to complete missing information on follow-up. The response to fecal diversion was evaluated by clinical examination, patient history, and endoscopy. If disease manifestations resolved completely (including an endoscopy demonstrating no residual inflammatory activity or stenosis), the response was classified as complete remission. If disease activity was improved but not completely resolved or if not all disease manifestations resolved (e.g., resolution of colitis, but anal fistula did not respond), the response was classified as partial remission.

## 3. Results

Twenty-nine patients who received a temporary fecal diversion for colorectal or perianal Crohn's disease were included in the study. Details of the study population are provided in Table 1. The majority of patients (84.6%) received anti-TNF medications without sufficient control of the colorectal or perianal disease. Most patients (21/29, 72.4%) had previous operations or endoscopic interventions for the same indication that finally led to the actual temporary stoma creation, meaning that temporary fecal diversion was not the first line treatment in the majority of patients.

A loop ileostomy was the standard procedure of temporary fecal diversion in 25/29 patients (86.2%); however, 4 patients with isolated proctitis or perianal Crohn's disease had a loop colostomy created (13.8%). In 20/29 patients (69.0%), stoma creation was performed by laparoscopic approach. 7/29 (24.1%) developed complications related to stoma creation (Table 1), with three patients (10.3%) requiring surgery.

Information on medical therapy during fecal diversion was available for 25 patients. 15/25 (60%) received anti-TNF-alpha antibodies; 4 of these (16%) were previously naïve to this therapy and received the first administration after fecal diversion was established.

Indications for temporary fecal diversion were refractory colitis (including severe inflammation, stenosis, and fistulas) in 14/29 (48.3%), ileitis (affecting the terminal ileum) in 4/29 (13.8%), and perianal Crohn's disease (fistulas, abscesses, and perianal sepsis) in 22/29 (75.9%), meaning that patients frequently had more than one indication. Actual combinations of indications for all patients are shown in Table 2. Nineteen patients (65.5%) had only one indication; the remainder had two or more.

The outcome of temporary fecal diversion for each indication and for the total study population is shown in Table 2. The response of Crohn's disease manifestations to temporary fecal diversion was complete remission in 4/29 (13.8%), partial remission in 12/29 (41.4%), no change in 7/29 (24.1%), and progress of disease activity in 6/29 (20.7%). So overall 16/29 (55.2%) showed a clinical response to fecal diversion. The median time to the maximum clinical response was 24 (2–85) weeks. Although response rates varied between different indications, patient numbers were too small to allow comparisons.

Ileostomy or colostomy reversal (restoration of intestinal continuity) was performed in 19 out of 25 patients (76%) who were available for long-term follow-up; duration of fecal diversion was in median 13.0 months (2–90). Ileostomy reversal was planned in the near future in further 2/25 (8%). In 4/25 (16%), stoma reversal was not possible due to disease activity.

Follow-up time of the 19 patients having their intestinal continuity restored was in median 21 (1–68) months (starting with stoma reversal). The majority of patients (15/19, 78.9%) needed further surgical therapies for a relapse of the same pathology that previously led to temporary fecal diversion. The time interval between ileostomy reversal and consecutive surgery was in median 18.5 months (0–51). In 10/19 (52.6%), these were colorectal resections such as colectomy or proctocolectomy; 7/19 (36.8%) finally had a definitive stoma created. In 3/19 (15.8%), resection of the ileum was necessary; 5/19 (26.3%) needed operations for perianal Crohn's disease, such as seton drainage, or abscess excision.

Regarding the whole concept of temporary fecal diversion, the success rate is related to the number of patients with sufficient long-term follow-up (*n* = 25, including stoma reversal and clinical course after reversal). Finally only 4/25 patients (16%) reached a stable (partial) remission after temporary fecal diversion without the need for further surgery (follow-up times after stoma reversal: 4, 33, 39, and 52 months, resp.), including one patient with perianal CD, one patient with ileitis, and two patients with a combination of perianal CD and colitis.

## 4. Discussion

Our experiences demonstrate that temporary fecal diversion can induce remission or at least substantial improvement of disease activity in the majority of patients with colorectal or perianal Crohn's disease, even if refractory to intensified medical therapy. The stomas created are indeed “temporary” as they are reversed in most patients in our series. However, most patients will develop a relapse of exactly the same disease manifestations that initially led to stoma creation, meaning that the concept of temporary fecal diversion fails in most patients. These patients finally need definitive surgical therapies including colorectal resections.

Compared to medical treatments, the response rate of about 55% is considerable, especially in the light of intensive previous medical therapy including anti-TNF-alpha antibodies in the majority of patients. The striking effect of fecal diversion on Crohn's disease activity was already described in the 60s and 70s [1–4]; in those days, stoma creation sometimes was the only chance to bring patients into an acceptable nutritional status and general condition that allowed extensive surgical resections at a later time point [2, 4]. Interestingly, the effect of fecal diversion on ulcerative colitis is disappointing [7], underlining the different characteristics of the two disease entities. It is not completely clarified how fecal diversion ameliorates Crohn's disease activity. Biochemical alterations of the colonic mucosa and modifications of fecal bacterial load seem to play a role [13, 14].

Many patients are concerned that the “temporary” stoma may not be reversed as initially intended. In our series, we considered stoma reversal whenever possible, especially if a substantial improvement or even remission of the underlying disease was reached. However, we informed all of our patients about the substantial risk of relapse. In our experience, most patients opted for an attempt of stoma reversal. Other authors seem to be somewhat more restrictive concerning stoma reversal, as most reports have lower rates of stoma reversal, ranging from 10 to 52% [6–12, 15]. Performing stoma reversal in patients who do not reach a complete remission of their colonic or perianal disease might partly explain the high rate of relapses following stoma reversal in our series.

It is important to note that relapses can occur even if the fecal diversion is maintained. While we did not observe such a relapse during fecal diversion as we reversed all stomas as soon as a stable response was reached, other studies demonstrated relapses at a median of 23 months in 30–60% of patients that initially responded to fecal diversion [5, 15].

The high relapse rate after stoma reversal is the critical topic that impairs the overall success rate of the concept of temporary fecal diversion. At the end of follow-up, only 16% of our patients needed no further stoma creation or colorectal resections; and even in these patients, disease activity again required intensified medical therapy. This disappointing success rate of temporary fecal diversion (including remission of disease, actual stoma reversal, and relapse-free course after stoma reversal) is comparable to other several studies that reported rates from 5 to 23% [8, 9, 12, 15].

This study was performed in the anti-TNF-alpha era, and most patients received anti-TNF-alpha medications before or during fecal diversion. Our data do not allow an evaluation of the possible advantage of these medications in the setting of temporary fecal diversion. However, as our success rate is as low as in older studies conducted in the pre-anti-TNF-alpha era [6–8, 15], anti-TNF-alpha medications seem not to substantially improve the outcome of this concept. This is underlined by a series of Uzzan et al. who combined ileal diversion and anti-TNF-alpha medications in patients with severe Crohn's colitis and perianal fistulas [16]. All three patients in that series finally needed a permanent stoma.

In the light of the high relapse rates after stoma reversal, the value of temporary fecal diversion for colonic and perianal Crohn's disease is questionable. The chance of enduring remission of the disease while avoiding extensive colorectal resection is rather small, and the need for two operations (stoma creation and reversal) leading to relevant morbidity has to be taken into account. Nevertheless, some patients will opt for this chance. Psychological aspects on behalf of the patient are of great importance. Specifically young patients often want to avoid definitive, irreversible resections as they are felt to be mutilating. In these cases, temporary fecal diversion is an effective way to “buy time” and to postpone the decision on definitive surgical treatments, at the same time providing the small chance that definitive surgical treatments might even be avoided at all. Many patients are afraid of definitive stoma creation. However, a recent study on patients with perianal Crohn's disease has shown that fecal diversion on the contrary has the potential to improve subjective quality of life [17]. Most of the patients in our series had undergone various medical therapies that were not able to relieve disease activity before fecal diversion was indicated. In our experience, most patients appreciate the great advantages of fecal diversion, especially the relief of symptoms. After several months of getting accustomed to the presence of a stoma, patients are more likely to accept the idea of carrying a definitive stoma.

## 5. Conclusions

Although temporary fecal diversion can induce remission in otherwise refractory colonic or perianal Crohn's disease, the chance of enduring remission after stoma reversal is very low and most patients will require definitive surgical therapies at a later stage. However, in selected patients, the concept of temporary fecal diversion still is an option, as it provides time buying before definitive surgery, allows accustoming the patient to the presence of a stoma, and has the potential to improve the conditions of definitive surgical treatment “bridge to definitive surgery”.

## Figures and Tables

**Table 1:** Patient characteristics, details of stoma creation, and medication.

Total number of patients	29
Male : female	15 : 14
Median age (years)	30.0 (range, 18–76)
Median disease duration (years)	7.0 (range, 0–32)
Follow-up time (months)	33.0 (range, 3–103)
Medication at fecal diversion	
Infliximab	14/26 (53.8%)
Adalimumab	8/26 (30.8%)
Azathioprine	16/26 (61.5%)
Methotrexate	5/26 (19.2%)
Mesalazine	3/26 (11.5%)
Steroids	10/26 (38.5%)
Type of fecal diversion	
Loop ileostomy	25/29 (86.2%)
Loop colostomy	4/29 (13.8%)
Complications of stoma creation	
Total	**7/29 (24.1%)**
Parastomal hernia	3/29 (10.3%)
Parastomal abscess	2/29 (6.9%)
Stoma prolapse	1/29 (3.4%)
Renal failure due to high output	1/29 (3.4%)
Medication during fecal diversion	
Infliximab	5/25 (20%)
Adalimumab	10/25 (40%)
Azathioprine	6/25 (24%)
Methotrexate	1/25 (4%)
Mesalazine	1/25 (4%)
Steroids	5/25 (20%)

**Table 2:** Outcome of fecal diversion for different indications.

Indication for fecal diversion	Patient number	Response to fecal diversion				Stoma reversal	Success (no further surgical therapy required)	Surgical therapy after stoma reversal			
		Complete remission	Partial remission	No change	Progress			Colorectal resections	Definitive stoma	Resection of ileum	Surgery for perianal CD
Perianal CD only	12/29 (41.4%)	3/12 (25.0%)	4/12 (33.3%)	4/12 (33.3%)	1/12 (8.3%)	8/10 (80.0%)	1/10 (10.0%)	4/8 (50.0%)	2/8 (25.0%)	0/8 (0.0%)	3/8 (37.5%)
Colitis only	6/29 (20.7%)	0/6 (0.0%)	3/6 (50.0%)	0/6 (0.0%)	3/6 (50.0%)	3/6 (50.0%)	0/6 (0.0%)	2/3 (66.7%)	3/3 (100.0%)	2/3 (66.7%)	1/3 (33.3%)
Ileitis only	1/29 (3.4%)	0/1 (0.0%)	1/1 (100.0%)	0/1 (0.0%)	0/1 (0.0%)	1/1 (100.0%)	1/1 (100.0%)	0/1 (0.0%)	0/1 (0.0%)	0/1 (0.0%)	0/1 (0.0%)
Colitis + perianal CD	7/29 (24.1%)	1/7 (14.3%)	3/7 (42.9%)	1/7 (14.3%)	2/7 (28.6%)	5/6 (83.3%)	2/6 (33.3%)	3/5 (60.0%)	2/5 (40.0%)	0/5 (0.0%)	1/5 (20.0%)
Ileitis + perianal CD	2/29 (6.9%)	0/2 (0.0%)	1/2 (50.0%)	1/2 (50.0%)	0/2 (0.0%)	1/1 (100.0%)	0/1 (0.0%)	0/1 (0.0%)	0/1 (0.0%)	1/1 (100.0%)	0/1 (0.0%)
Colitis + ileitis + perianal CD	1/29 (3.4%)	0/1 (0.0%)	0/1 (0.0%)	1/1 (100.0%)	0/1 (0.0%)	1/1 (100.0%)	0/1 (0.0%)	1/1 (100.0%)	0/1 (0.0%)	0/1 (0.0%)	0/1 (0.0%)
Total	**29 (100%)**	**4/29 (13.8%)**	**12/29 (41.4%)**	**7/29 (24.1%)**	**6/29 (20.7%)**	**19/25 **(76.0%)^a^	**4/25 **(16.0%)^b^	**10/19 **(52.6%)^c^	**7/19 **(36.8%)^c^	**3/19 **(15.8%)^c^	**5/19 **(26.3%)^c^

Indications (including combinations of indications) for stoma creation are listed. For each indication, response to diversion, rate of stoma reversal, and outcome are reported.

^a^Due to missing follow-up data of 4 patients, only 25 patients were available for the criterion “stoma reversal.”

^b^The total success rate is related to all patients with sufficient follow-up (*n* = 25, including patients in whom the stoma was not reversed).

^c^The sum of percentages of definitive surgical therapies exceeds 100%, as patients frequently had more than one type of surgery.

CD: Crohn's disease.
